# Enhancement of Luminance in Powder Electroluminescent Devices by Substrates of Smooth and Transparent Cellulose Nanofiber Films

**DOI:** 10.3390/nano11030697

**Published:** 2021-03-10

**Authors:** Shota Tsuneyasu, Rikuya Watanabe, Naoki Takeda, Kojiro Uetani, Shogo Izakura, Keitaro Kasuya, Kosuke Takahashi, Toshifumi Satoh

**Affiliations:** 1 Department of Media Engineering, Graduate School of Engineering, Tokyo Polytechnic University, 1583 Iiyama, Atsugi, Kanagawa 243-0297, Japan; s.tsuneyasu@mega.t-kougei.ac.jp (S.T.); m1716089@st.t-kougei.ac.jp (R.W.); naoki.takeda@toppan.co.jp (N.T.); 2 The Institute of Scientific and Industrial Research (SANKEN), Osaka University, Mihogaoka 8-1, Ibaraki, Osaka 567-0047, Japan; uetani@eco.sanken.osaka-u.ac.jp; 3 Graduate School of Engineering, Osaka University, Mihogaoka 8-1, Ibaraki, Osaka 567-0047, Japan; u895635a@gmail.com (S.I.); keitaro_k@chem.eng.osaka-u.ac.jp (K.K.); h.takahashi@eco.sanken.osaka-u.ac.jp (K.T.)

**Keywords:** powder electroluminescent device, cellulose nanofiber, paper electronic device, paper-based light-emitting device

## Abstract

Powder electroluminescent (EL) devices with an electric field type excitation are surface light sources that are expected to have a wide range of practical applications, owing to their high environmental resistance; however, their low luminance has hindered their use. A clarification of the relationship between the properties of the film substrates and the electroluminescence is important to drastically improve light extraction efficiency. In this study, powder EL devices with different substrates of various levels of surface roughness and different optical transmittances were fabricated to quantitatively evaluate the relationships between the substrate properties and the device characteristics. A decrease in the surface roughness of the substrate caused a clear increase in both the current density and the luminance. The luminance was found to have a direct relationship with the optical transmittance of the substrates. The powder EL device, which was based on a cellulose nanofiber film and was the smoothest and most transparent substrate investigated, showed the highest luminance (641 cd/cm^2^) when 300 V was applied at 1 kHz.

## 1. Introduction

The development of a flexible flat light-emitting device that includes organic and inorganic light-emitting devices beyond the traditional solid and rigid displays of liquid crystal or light-emitting diodes is useful for the establishment of a next-generation super-smart society. A powder electroluminescent (EL) device is a traditional flexible flat light source that involves inorganic EL particles and can be fabricated using a simple printing system on a film substrate, such as copy paper and plastic films. Compared with the conventional organic flat light-emitting devices with a current-type excitation (which are very sensitive to the surrounding environment (i.e., water and oxygen)), the powder EL device has a high environmental resistance, owing to its characteristic electric field type excitation. Because of this advantage, the powder EL devices, which can be fabricated by 3D printing [1,2], exhibit good mechanical durability, such as bending durability [3], flexibility [4], and stretchability [5,6]. They can also be used as sensors for ammonia [7], carbon dioxide [8], humidity [9], and temperature [10]. Unique functionalities, such as the self-recovery of their structure [11,12,13] and the generation of sound [14,15,16], have also been developed through the powder EL system. However, the practical use of powder EL devices has only been based on indirect illumination, owing to their low luminance. For a long time, organic light emitting devices were thought to have difficulty in achieving laser oscillation due to their low brightness. Recently, the laser action demonstrated in organic semiconductors by the distributed feedback structure has exhibited optical confinement effects [17]. If the luminance in powder EL devices with low luminance can be significantly improved, the desired laser action might be observed by introducing the rib and slot-waveguide structures [18,19]. The intrinsic improvement of the luminance for a powder EL device is the key to develop flexible and versatile light-emitting devices.

In terms of achieving high luminance from a powder EL device, researchers have focused their investigations on the phosphor [20] and the high dielectric polymer [21] components. Additionally, a high luminance has been achieved by drastically modifying the device structure [22,23]. However, we previously focused on the surface roughness of the paper substrate and found that the planarization of the substrate is effective to achieve high luminescence from a bottom emission-type powder EL device [24]. Therefore, there is great potential to improve EL luminance by investigating the properties of the film substrates. However, the direct effect of the film properties (which include surface roughness and transparency) on EL luminance has not yet been clarified. This is because EL luminance is complex and is affected by multiple factors, such as applied voltage and frequency. Furthermore, powder EL devices with a paper substrate have often employed a top emission-type structure that is unaffected by the optical transmittance of the substrate films [25,26,27]. To essentially improve the light extraction efficiency in powder EL devices, the direct effect of the properties of the film substrates needs to be investigated by using a bottom emission-type device.

Recently, cellulose nanofiber (CNF) films have been developed from various phytomass resources and have been shown to exhibit high transparency [28,29] and minimal surface roughness [30,31], as well as good flexibility. Compared with conventional plastics, CNF films are also known to display high strength [32,33] and thermal stability (a low coefficient of thermal expansion) [34,35], owing to the extended chain crystals of natural cellulose type I. This CNF film is a “paper” material in which the CNFs selfagglutinate through the strong hydrogen bonds on their surface. Therefore, the CNF film is proposed to be the most legitimate successor of a conventional paper substrate in powder EL devices, owing to its high performance.

In this study, we aimed to elucidate the effects of surface roughness and the transmittance of the film substrates on the luminance of powder EL devices and achieve a high luminance that exceeds that of the devices composed of conventional paper and plastic film substrates. Powder EL devices were fabricated with four different substrates: tracing paper, polyethylene naphthalate (PEN) film, commercial CNF (C-CNF) sheets, and 2,2,6,6-tetramethylpiperidine-1-oxyl (TEMPO)-oxidized CNF (TO-CNF) films. Each device had a different surface roughness and optical transmittance, and the luminance performances of each device were compared.

## 2. Materials and Methods

### 2.1. Materials

Tracing paper (Sakae Technical Paper Co., Ltd., Tokyo, Japan), the C-CNF sheets (WMa-100FM, Sugino Machine Ltd., Toyama, Japan), and the PEN film (Q65FA, Teijin DuPont Films, Tokyo, Japan) were purchased and used as received.

The TO-CNF film was produced according to the previously reported method. In brief, Japanese cedar (Cryptomeria japonica) chips (50 g) were dewaxed by immersion in 1 L of acetone overnight. After removing the acetone, the resulting product was bleached in 1.5 L of 1.0 wt.% NaClO_2_ solution under acidic conditions at 80–90 °C for 6 h until the product became white, in accordance with Wise’s method [36]. The product was then washed with distilled water to obtain wood pulp. The pulp (with a dry weight of 5 g) was dispersed in 500 mL of distilled water and 0.08 g of TEMPO with the addition of 0.5 g of NaBr [37]. Oxidation was initiated by adding 27 mL of 1.8 M NaClO aqueous solution, and the pH was kept higher than 10 by adding 0.5 M NaOH. After reacting for 7 h, the oxidation was quenched by adding 5 mL of ethanol and then washed until the pH reached ~7 [38]. The product was redispersed in 500 mL of distilled water, further treated by adding 5.0 g of NaClO_2_ under acidic conditions, and stirred for 48 h to selectively convert the aldehyde group in the samples to a carboxylate group [39].

After washing, a TEMPO-oxidized pulp with a carboxylate content of 1.67 mmol/g (determined via conductometric titration) was obtained. The TEMPO-oxidized pulp was preliminarily agitated at 24,000 rpm by a high-speed blender for 10 min and was then passed through a high-pressure water jet system (Star Burst 10, HJP-25008, Sugino Machine Ltd., Toyama, Japan) at 180 MPa for 50 cycles to produce an aqueous suspension of TEMPO-oxidized cellulose nanofibers. After removing the bubbles, the viscous nanofiber suspension (with a concentration of 1.5 wt.%) was cast on an acrylic plate by using a metal formwork with internal dimensions of 7 × 7 cm^2^ and a thickness of 2 mm, and this cast was dried in an oven at 55 °C overnight to form the TO-CNF films.

Zinc sulfide-type particles (GG45, Osram Sylvania, Wilmington, MA, USA) were used as a phosphor layer, barium titanium oxide (620-07545, Kishida Chemical Co., Ltd., Osaka, Japan) was used as a dielectric layer, cyanoethyl polyvinyl alcohol (cyanoresin, CR-V, Shinetsu Chemical Co., Ltd., Tokyo, Japan) was used as a high dielectric polymer, poly(2,3-dihydrothieno-1,4-dioxin)–poly(styrenesulfonate) (PEDOT:PSS, 768650, Sigma-Aldrich Co., LLC., Tokyo, Japan) was used as a transparent electrode, and Ag-paste (MP-603S, Mino Group Co., Ltd., Gifu, Japan) was used as a back electrode, all as received. Cyclohexanone (037-05096) was purchased from Fujifilm Wako Pure Chemical Co., Osaka, Japan.

### 2.2. Characterization of the Substrate Films

The regular light transmittance was measured with a spectrophotometer (U-3900, Hitachi High-Tech Corp., Tokyo, Japan). The haze was evaluated using a haze meter (HZ-V3, Suga Test Instruments Co., Ltd., Tokyo, Japan). The surface roughness (the root mean square (RMS)) was determined for an area of 20 × 20 µm^2^ using an atomic force microscope (AFM, Nanocute, SII Nano Technology Inc., Chiba, Japan) in dynamic force mode [40,41]. The three-dimensionally extended RMS of cross-sectional curves defined in JIS B 0601 (ISO 4287) was calculated for the measured surfaces, and it is expressed as the square root of the average of the square of deviation from the reference surface to the specified surface. In this study, RMS was calculated for the entire 20 × 20 µm^2^ angle of view.

### 2.3. Fabrication of the EL Devices

The cyanoresin was mixed in cyclohexanone at 30 wt.% to prepare the high-dielectric polymer paste. Then, 40 wt.% of a zinc sulfide-type phosphor was dispersed in the polymer paste. Barium titanium oxide was also dispersed in the polymer paste, in the same weight ratio as the phosphor had been dispersed. The transparent electrode, phosphor layer, dielectric layer, and back electrode were laminated by an automatic screen-printing machine (TU2020-C, Seritech Co., Ltd., Osaka, Japan) on each substrate in this order. Each layer was individually dried in an oven at 80 °C for 6 min; the transparent electrode layer was dried for 10 min at 80 °C to fully evaporate the extra solvents. The light-emitting area of the devices was set as 1.0 × 1.0 cm^2^. The cross-sectional layer structure for each EL device was observed by an optical microscope (WPA-micro, Photonic Lattice Inc., Miyagi, Japan).

### 2.4. EL Measurements

The voltage dependences of the current and the luminance of the devices were determined by using an EL measurement system (SX-1152, Iwatsu Electric Co., Ltd., Tokyo, Japan).

## 3. Results and Discussion

**Figure 1:**
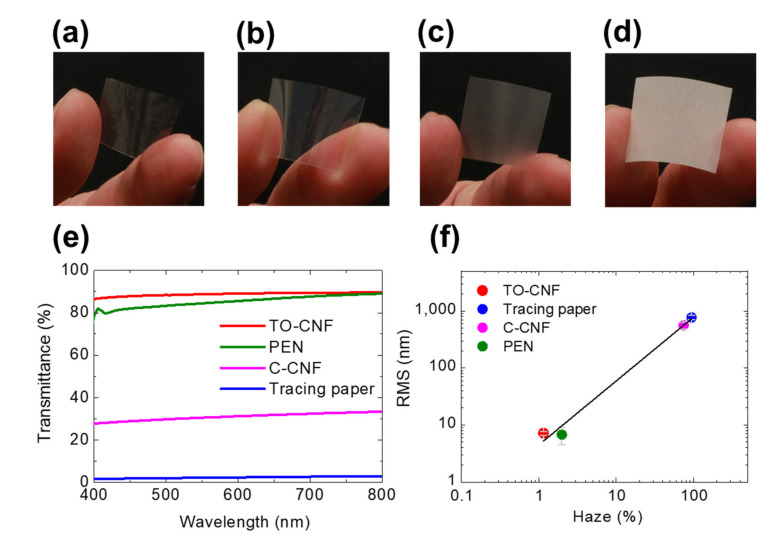
Photographs of (**a**) 2,2,6,6-tetramethylpiperidine-1-oxyl (TEMPO)-oxidized cellulose nanofiber (CNF) (TO-CNF), (**b**) polyethylene naphthalate (PEN), (**c**) commercial CNF (C-CNF), and (**d**) tracing paper films. (**e**) Transmittance spectra of each film. (**f**) The relationship between root mean square (RMS) roughness and haze.

The regular light transmittance of the four films was investigated. Figure 1a–e shows a photograph of and the optical transmittance of each film. The TO-CNF, PEN, and C-CNF films appeared highly transparent, as shown in Figure 1a–c, respectively; however, each of these films had a different transmittance spectrum (Figure 1e). The TO-CNF film showed the highest transparency among the four, and the transmittance in the visible light range for this film reached approximately 90%. The PEN film showed almost the same transmittance as the TO-CNF film at wavelengths greater than 700 nm, but the transmittance gradually decreased at lower wavelengths. The C-CNF film had a transmittance of ~30% over a wide wavelength range. The tracing paper was completely opaque with the lowest transmittance of below 3%, as shown in Figure 1d, but it has long been used as a powder EL substrate in previous studies [10,24].

**Figure 2:**
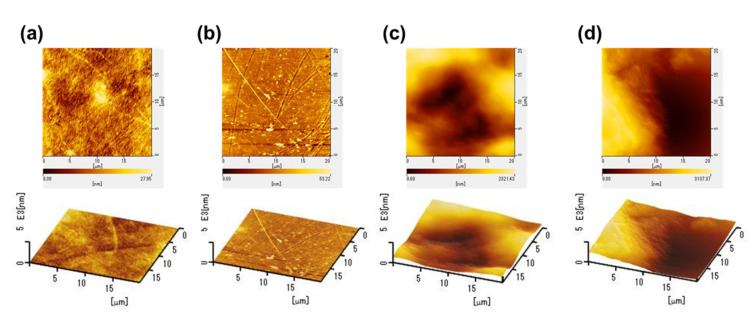
Atomic force microscope (AFM) images of (**a**) TO-CNF, (**b**) PEN, (**c**) C-CNF, and (**d**) tracing paper substrates for the calculation of RMS roughness.

We evaluated the surface roughness of each film using the RMS values obtained from the AFM measurement (Figure 2). The RMS roughness of each of the TO-CNF, PEN, C-CNF, and tracing paper films were found to be around 6.78 ± 2.3 nm, 7.19 ± 0.54 nm, 560.9 ± 72.7 nm, and 769 ± 64.5 nm, respectively (as shown in Table 1). We found that the film with a smaller RMS value showed a higher transmittance. To evaluate the optical properties, the haze of each film was also measured. The haze of the TO-CNF, PEN, C-CNF, and tracing paper substrates were approximately 1.99 ± 0.06%, 1.15 ± 0.10%, 74.34 ± 0.41%, and 93.96 ± 0.04%, respectively. We obtained a linear relationship between the RMS values and haze, as shown in Figure 1f. The surface roughness of each film was shown as directly related to its optical transparency. The negative linear correlation between RMS and transparency in films was also found for microcrystalline diamond [42]. In general, it is also empirically self-evident that a rough surface leads to opacity, as seen in frosted glass. Regardless of the chemical composition, the transparency of the film is strongly correlated with the surface roughness, which is also shown in this study.

The powder EL devices were fabricated using these films as substrates. To explore the electrode distance of each device, the cross-section of each EL device was observed using an optical microscope (Figure S1). The thickness of the functional layers of the devices with the TO-CNF, PEN, C-CNF, and tracing paper substrates were measured to be 106.0 ± 2.3 μm, 129.4 ± 3.8 μm, 94.0 ± 3.6 μm, and 122.6 ± 6.7 μm, respectively, although the thicknesses of the film substrates were significantly different. It was therefore suggested that almost the same electric field intensity could be obtained when the same voltage was applied, regardless of the type of substrate film.

**Figure 3:**
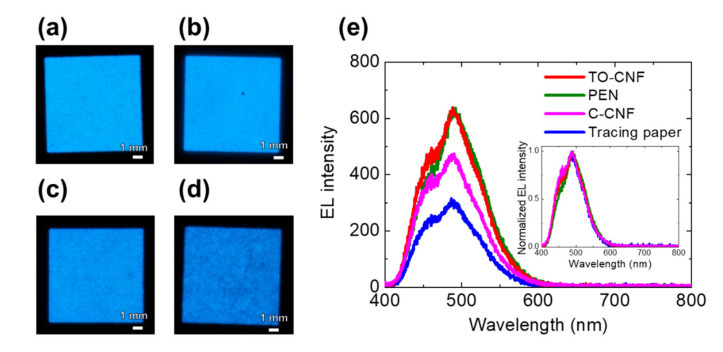
Photographs of the powder EL devices with a substrate of (**a**) TO-CNF, (**b**) PEN, (**c**) C-CNF, and (**d**) tracing paper under the application of an AC voltage of ±170 V at 1.2 kHz. (**e**) Electroluminescent (EL) spectra of the devices with each substrate under the application of an AC voltage of ±170 V at 1.2 kHz. The inset shows the normalized EL spectra of the devices.

Figure 3a–d shows photographs of the luminescence of each EL device with a different substrate film under the application of an AC voltage of ±170 V at 1.2 kHz. All of the EL devices emitted blue light. The emissions from the devices with C-CNF and tracing paper substrates were uneven and dark. This was proposed to arise from the variation of the electrode distance, which could not be detected from local observation with an optical microscope. The variation of the electrode distance occurred owing to the large surface roughness of the substrate, which led to a variation in the electric field intensity. The EL spectrum of these devices was investigated to ascertain the effect of the substrates on EL emission. The EL intensity of the devices with the TO-CNF and PEN substrates under application of the same voltage was higher than those with the C-CNF and tracing paper substrates (Figure 3e). This was considered to arise from the differences in the surface roughness and optical transmittance of the substrates. As shown in the inset of Figure 3e, the EL band with an emission peak was observed at a wavelength of 490 nm, which was almost identical to that observed from previous devices [24].

**Figure 4:**
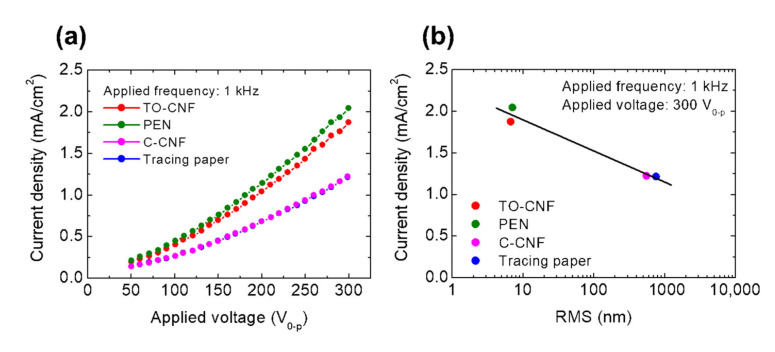
EL property measurement. (**a**) The relationship between current density at an applied frequency of 1 kHz and applied voltage. (**b**) The relationship between current density at an applied voltage of ±300 V_0__–p_ at 1 kHz and the RMS roughness of the substrate films.

To clarify the EL properties, the voltage dependence of the current density of the EL devices with each substrate was measured (Figure 4a). We set the current density at 1 kHz because the commercialized zinc sulfide-type particles (GG45, Osram Sylvania) used in this study are known to give the best luminescence efficiency at 1 kHz [43]. The current density of all devices increased as the applied voltage increased. The current density of the devices with the TO-CNF and PEN substrates was significantly higher than those with the C-CNF and tracing paper substrates. As shown in Figure 4b, under application of an AC voltage of ±300 V at 1 kHz, the current density exponentially decreased as the RMS roughness of the substrate film was increased. Thus, the current density of the powder EL device was found to be dependent on the RMS roughness of its substrate.

**Figure 5:**
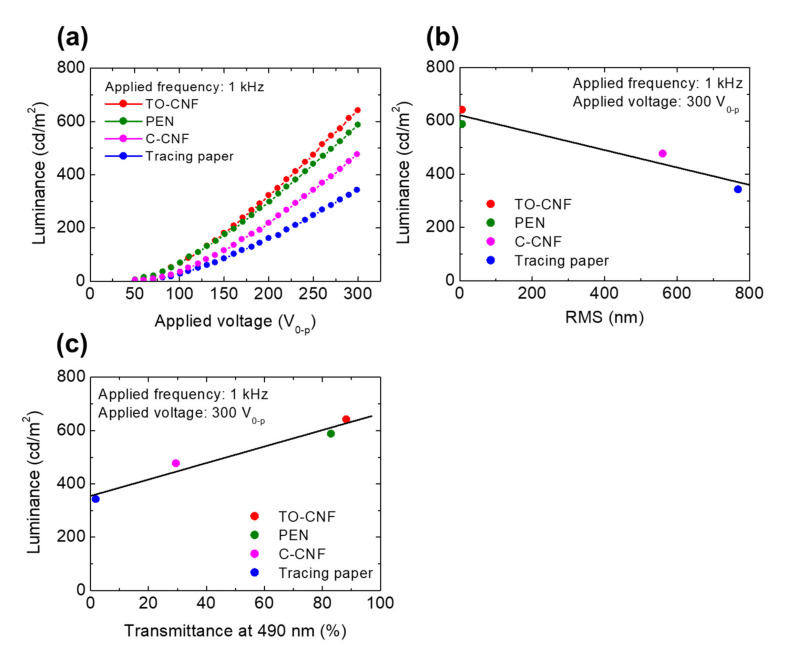
(**a**) The luminance of the EL devices versus the applied voltage characteristics at an applied frequency of 1 kHz. The luminance of the EL devices at an applied voltage of 300 V_0__–p_ at 1 kHz versus (**b**) RMS roughness and (**c**) transmittance of each substrate at a wavelength of 490 nm.

The applied voltage dependence of the luminance of the EL devices with each substrate was measured (Figure 5a). By scanning the applied voltage from ±50–300 V, the luminance of all devices increased with the increase of the applied voltage. Under the application of ±300 V at 1 kHz, the luminance from the device with the TO-CNF substrate was approximately 641 cd/m^2^ and was the highest compared with those of the devices with the other substrates. Compared with previous reports on bottom emission-type EL devices, which showed a maximum luminance of 150 cd/m^2^ under application of an AC voltage of ±150 V at 0.4 kHz [44], our 641 cd/m^2^ demonstrated a drastic enhancement of the luminance for the bottom emission-type EL device with a paper substrate.

To quantitatively elucidate the relationship between the substrate characteristics and the obtained luminance, the RMS roughness of the film substrates and their EL luminance under application of an AC voltage of ±300 V at 1 kHz is plotted in Figure 5b. The luminance linearly increased with a decrease in RMS roughness in a similar manner as the current density described above in Figure 4b. Thus, the current density can be increased by an improvement of the RMS roughness of the substrates, which will lead to a high luminance in powder EL devices. Figure 5c shows the clear relationship between the transmittance of the substrate films at 490 nm (which is the wavelength of the emission peak) and the luminescence of the EL devices. The luminance linearly increased with an increase of the optical transmittance of the substrates. It is now clearly demonstrated that the required traits of a substrate for obtaining high luminance in a powder EL device are not only a smooth surface but also a high transparency. These findings are key factors for suitably designing the luminance in powder EL devices.

## 4. Conclusions

We have clarified the effect of the physical state of the film substrate (surface roughness and transparency) on the luminance of powder EL devices, which has not been focused on before. At the same time, we have succeeded in producing the powder EL device with the highest luminance of 641 cd/cm^2^ when 300 V was applied at 1 kHz by using the substrate of cellulose nanofiber film, a next-generation paper material with high smoothness, transparency, and other excellent properties.

A typical application of powdered EL devices is as a surface emitting light source, and displays and lighting are its most common applications. The present study, which clarified the physical guideline for improving the luminance (the lighting performance) using natural materials that promote the elimination of plastics as the base material, is expected to provide a significant guideline for the development of next-generation high-performance lighting. We believe that the findings of this research will be useful in the future for highly linear point light sources because the device might achieve a laser oscillation by the introduction of rib and slot-waveguides structures.

## Figures and Tables

**Table 1:** The characteristics of each film and the thickness of the functional layer on those devices.

	Film Thickness (μm)	RMS Roughness (nm)	Haze (%)	Thickness of the Functional Layers (μm)
TO-CNF	22.0 ± 2.0	6.78 ± 2.3	1.99 ± 0.06	106.0 ± 2.3
PEN	128.0 ± 2.8	7.19 ± 0.54	1.15 ± 0.10	129.4 ± 3.8
C-CNF	54.0 ± 0.4	560.9 ± 72.7	74.34 ± 0.41	94.0 ± 3.6
Tracing paper	82.0 ± 1.4	769 ± 64.5	93.96 ± 0.04	122.6 ± 6.7

## Supplementary Materials

The following are available online at https://www.mdpi.com/2079-4991/11/3/697/s1, Figure S1: Cross-sectional optical microscopy images of (a) TO-CNF, (b) PEN, (c) C-CNF, and (d) tracing paper substrates.
