# Supplementary material for: Enhancement of Luminance in Powder Electroluminescent Devices by Substrates of Smooth and Transparent Cellulose Nanofiber Films
Source: Nanomaterials (Basel). 2021 Mar 10;11(3):697. doi: 10.3390/nano11030697 (PMC8001356; doi:10.3390/nano11030697)
Supplement: Supplementary file 1 [file nanomaterials-11-00697-s001.pdf]

nanomaterials

Supplementary Materials

Enhancement of Luminance in Powder Electroluminescent Devices by Substrates of Smooth and Transparent Cellulose Nanofiber Films

Shota Tsuneyasu [1], Rikuya Watanabe [1], Naoki Takeda [1], Kojiro Uetani [2], Shogo Izakura [3], Keitaro Kasuya [3], Kosuke Takahashi [3] and Toshifumi Satoh [1,*]

[1] Department of Media Engineering, Graduate School of Engineering, Tokyo Polytechnic University, 1583 Iiyama, Atsugi, Kanagawa 243-0297, Japan; s.tsuneyasu@mega.t-kougei.ac.jp (S.T.); m1716089@st.t-kougei.ac.jp (R.W.); naoki.takeda@toppan.co.jp (N.T.)
[2] The Institute of Scientific and Industrial Research (SANKEN), Osaka University, Mihogaoka 8-1, Ibaraki, Osaka, 567-0047, Japan; uetani@eco.sanken.osaka-u.ac.jp
[3] Graduate School of Engineering, Osaka University, Mihogaoka 8-1, Ibaraki, Osaka 567-0047, Japan; u895635a@gmail.com (S.I.); keitaro_k@chem.eng.osaka-u.ac.jp (K.K.); h.takahashi@eco.sanken.osaka-u.ac.jp (K.T.)
* Correspondence: toshi@mega.t-kougei.ac.jp

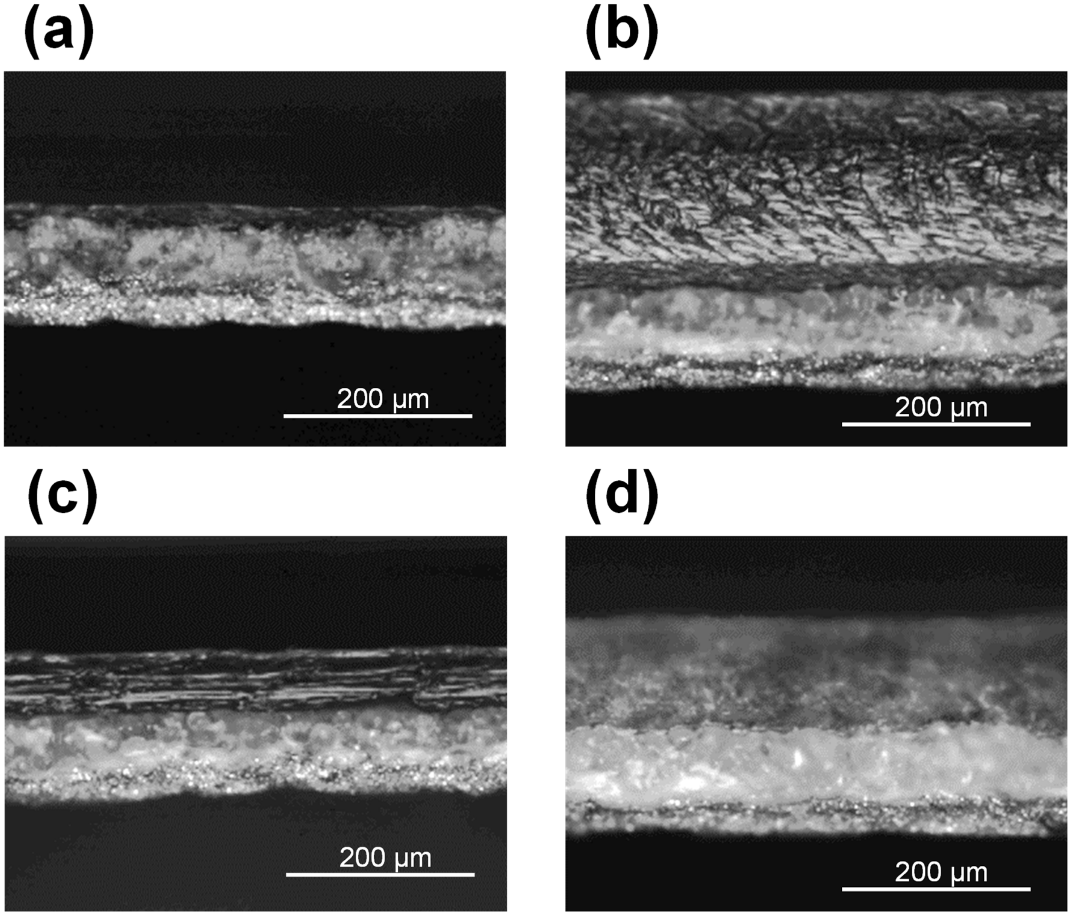

Figure S1. Cross-sectional optical microscopy images of (**a**) TO-CNF, (**b**) PEN, (**c**) C-CNF, and (**d**) tracing paper substrates.
